# Molecular Profiling of Endocrine Resistance in HR+/HER2-Metastatic Breast Cancer: Insights from Extracellular Vesicles-Derived DNA and ctDNA in Liquid Biopsies

**DOI:** 10.3390/ijms252313045

**Published:** 2024-12-04

**Authors:** Ana Martínez-Rodríguez, Jesús Fuentes-Antrás, Víctor Lorca, Alfonso López de Sá, Pedro Pérez-Segura, Fernando Moreno, Jose Angel García-Sáenz, Vanesa García-Barberán

**Affiliations:** 1 “Clinical and Translational Research in Oncology” Group, Molecular Oncology Laboratory, Hospital Clinico San Carlos, Instituto de Investigación Sanitaria Hospital Clinico San Carlos (IdISSC), 28040 Madrid, Spain; amartinez.rodriguez@salud.madrid.org (A.M.-R.);; 2 Department of Medical Oncology, Hospital Clinico San Carlos, Instituto de Investigación Sanitaria Hospital Clinico San Carlos (IdISSC), 28040 Madrid, Spain; 3 NEXT Oncology Experimental Therapeutics Unit, Hospital Universitario Quironsalud Madrid, 28223 Madrid, Spain

**Keywords:** metastatic breast cancer, endocrine resistance, liquid biopsy, extracellular vesicles, ctDNA, *PIK3CA*, *ESR1*

## Abstract

Standard treatments in hormone receptor-positive (HR+)/HER2-metastatic breast cancer (mBC) typically involve endocrine therapy (ET) combined with CDK4/6 inhibitors, yet resistance to ET remains a persistent challenge in advanced cases. A deeper knowledge of the use of liquid biopsy is crucial for the implementation of precision medicine in mBC with real-time treatment guidance. Our study assesses the prognostic value of *PIK3CA* and *ESR1* mutations in DNA derived from extracellular vesicles (EV-DNA) in longitudinal plasma from 59 HR+/HER2-mBC patients previously exposed to aromatase inhibitors, with a comparative analysis against circulating tumor DNA (ctDNA). Mutations were evaluated by digital PCR. *PIK3CA* and *ESR1* mutations were found in 22 and 25% of patients. Baseline *ESR1* mutations in EV-DNA were associated with shorter progression-free survival (PFS) across the cohort, with the Y537S mutation showing a particularly strong impact on the outcome of fulvestrant-treated patients. In contrast, *PIK3CA* mutations in EV-DNA did not significantly correlate with PFS, whereas in ctDNA, they were linked to poor outcomes. Altogether, this study positions EV-DNA as a valuable biomarker alongside ctDNA, enriching the understanding of different analytes in liquid biopsy and supporting strategies for HR+/HER2-mBC in precision oncology.

## 1. Introduction

Breast cancer is the most common cancer among women in most countries worldwide, with an estimated 2.3 million cases diagnosed globally in 2022 [1]. HR+/HER2-breast cancer is the most common type, accounting for about 70% of all cases [2]. Unfortunately, a substantial proportion of these patients develop metastatic breast cancer, which is generally considered incurable [3]. Upfront lines of palliative systemic therapy typically involve the use of endocrine therapy (ET) (i.e., aromatase inhibitors, AIs; selective estrogen receptor degraders, SERDs) usually in combination with CDK4/6 inhibitors (CDK4/6i). ET resistance, however, presents a major challenge in managing the disease in the metastatic setting. Precision medicine approaches have led to the identification of several new targets for breast cancer treatment, including alterations in the PI3K signaling pathway and *ESR1* mutations, which have been associated with ET resistance [4]. Studies in breast cancer suggest that *PIK3CA* mutations are driver alterations and early events in the malignant transformation of these tumors. They are present in 20–40% of all breast cancers, with the highest prevalence observed in luminal tumors. [5]. On the other hand, mutations in the *ESR1* gene have been linked to resistance to hormonal therapy, mainly as acquired resistance after treatment with aromatase inhibitors (AI), resulting in ligand-independent estrogen receptor activity. *ESR1* mutations can be detected in 20–50% of luminal cases previously treated with hormonal therapy, depending on the duration and type of therapy administered [6]. These mutations contribute to disease progression, prompting the development of specific drugs to improve patient survival. Some oral α-specific PI3K inhibitors have exhibited efficacy in clinical trials and were recently approved for use in combination with fulvestrant +/−palbociclib in these *PIK3CA* mutated, endocrine-resistant patients [7,8]. Other agents, such as capivasertib, an oral AKT inhibitor, have shown activity in PIK3–AKT–mTOR-activated pathway tumors [9]. Additionally, mutations in *ESR1* have been associated with a poorer response to treatment with AIs [10]. Thus, the PADA1 study showed that modifying the treatment regimen from an aromatase inhibitor in combination with palbociclib to fulvestrant plus palbociclib to address *ESR1* mutations improves clinical outcomes in this subgroup of patients [11].

In this context, liquid biopsy has become a crucial step toward the implementation of precision medicine, enabling a better understanding of cancer molecular profiling and the resistance pathways in real-time [12], being incorporated into several clinical trials to optimize early detection, treatment selection, and monitoring, and even in detecting minimal residual disease [13,14,15,16]. Different analytes found in liquid biopsies hold the potential to reveal insights into tumor characteristics [17]. One such analyte is cell-free DNA (cfDNA), which is released into the bloodstream through cell death or active secretion, encompassing a range of DNA types circulating at any given time. Among these, a portion comprises circulating tumor DNA (ctDNA) originating from tumor cells. Consequently, plasma ctDNA provides a non-invasive way to access the molecular content of cancer cells, and its role as a biomarker is currently widely studied. Additionally, the presence of a relatively abundant background of wild-type DNA underscores the need for sensitive methodologies, where the assessment of DNA contained in extracellular vesicles (EVs) could potentially improve test performance. EVs, including exosomes, are small, membrane-bound vesicles secreted by most cell types, including cancer cells, into the extracellular environment. These vesicles enter the bloodstream and carry diverse genetic material, including DNA, protected from enzymatic degradation by their lipid bilayer [18]. DNA isolated from EVs (EV-DNA) originates from nuclear, mitochondrial, or cytoplasmic sources and is released into the extracellular space through both passive mechanisms, linked to cell death processes, such as apoptosis and necrosis, and active secretion, which involves vesicle biogenesis in living cells [19]. Exosomes have been identified as valuable sources of biomarkers for breast cancer, with a primary focus on microRNAs (miRNAs) as prognostic and diagnostic biomarkers [20,21,22]. In the context of HR+/HER2-breast cancer, exosomes can transfer miRNAs that target genes involved in ET sensitivity [23,24]. Studies currently describe exosomes as a heterogeneous population of vesicles with varying properties and biogenesis pathways, but their definitive characterization remains challenging [25]. For this reason, the International Society for Extracellular Vesicles (ISEV) recommends the term “extracellular vesicles” (EVs) refers to lipid bilayer-enclosed particles released from cells that cannot replicate on their own [26].

In a previous study, we characterized longitudinal ctDNA and its role as a prognostic biomarker in HR+/HER2-mBC patients receiving endocrine-based therapies after being exposed to AIs in either the adjuvant or metastatic setting. The patients received ET with either an AI or a selective estrogen receptor degrader (SERD), with or without additional targeted agents such as CDK4/6i, and were monitored through liquid biopsies at various time points [27]. In this article, we focused on a subset of these patients to assess the prognostic value of EV-DNA. Additionally, we aimed to compare the tumor mutational information provided by EV-DNA and ctDNA.

## 2. Results

### 2.1. Study Population

Fifty-nine patients diagnosed with HR+/HER-mBC were enrolled in the study to analyze the *PIK3CA* and *ESR1* mutation status (Table 1). The median age of patients at enrollment was 65 years, ranging from 39 to 87 years. Only 13 patients presented de novo disease at diagnosis. For the remaining patients, the median time from the initial diagnosis to the first evidence of metastasis was 7.9 years (1.1–43), and enrollment was 9.41 years (0.3–52.5). At enrollment, 56% of patients had visceral metastatic disease, with liver involvement in 23%, lung involvement in 32%, and pleura involvement in 15%. None of the patients had central nervous system (CNS) involvement at baseline. Some patients presented bone-only disease (22%) or node-only disease (8%).

Regarding previous lines of ET in the metastatic setting, 7 patients (12%) had received none, 21 patients (36%) had received one, and 31 patients (52%) had received two or more prior lines of ET. Additionally, nine patients (15%) had received a prior line of non-adjuvant chemotherapy. Before enrollment, 39% of patients received therapy with CDK4/6i. Primary and secondary resistance criteria were met in a similar percentage of patients (29 and 25%, respectively). After enrollment, 71% of patients received SERD-containing regimens, 29% of patients received regimens containing AI, and 23% of patients received a concurrent CDK4/6i. The median follow-up period for study participants was 21.3 months (0.5–76.2). Thirty-five patients (57%) had died at study completion.

### 2.2. EV-DNA as a Source of Information on Tumor Mutational Status

Plasma samples were obtained from 59 patients at T0 to analyze with dPCR the following hotspot mutations in EV-DNA: *PIK3CA* E545K, E542K, H1047R, and *ESR1* Y537S and D538G (Supplementary Materials Figure S1). At baseline, the *PIK3CA* mutations exhibited a prevalence of 22.03% (13/59), with all mutated cases being monoclonal and a VAF ranging from 0.15% to 10.17% (median VAF: 1.12%). The mutation rates for E542K, E545K, and H1047R were 5.08% (3/59), 11.86% (7/59), and 5.08% (3/59), respectively. Variability in VAFs among positive cases was observed, with median VAFs of 3.45% (0.98–7.92%) for E542K, 0.66% (0.15–5.91%) for E545K, and 1.94% (1.26–10.17%) for H1047R. Regarding *ESR1*, a mutation rate of 25.42% (15/59) was observed, with all mutated cases being monoclonal and considerable variability in VAF values ranging from 0.08% to 21.9% (median VAF: 0.9%). For the Y537S mutation, a mutation rate of 10.17% (6/59) was observed, with a median VAF among positive cases of 0.98% (0.24–10.33%). Similarly, the D538G mutation showed a prevalence of 15.25% (9/59) at a median VAF of 0.9% (0.08–21.9%). Notably, five patients (8.5%) displayed coexisting *PIK3CA* and *ESR1* mutations.

Potential associations between mutational status in EV-DNA and the clinicopathological parameters were analyzed (Table 1). A trend towards a significant association was observed between *PIK3CA* mutations and previous treatment with CDK4/6i (*p* = 0.059). Additionally, *PIK3CA* mutations were more frequently detected in patients with bone-only metastases (*p* = 0.038). Patients with *ESR1* mutations in EV-DNA showed a higher likelihood of developing visceral metastases compared to those with wild-type *ESR1* (*p* < 0.001). No significant association was found between *ESR1* mutations and increased prior exposure to ET.

### 2.3. Dynamic Evolution of Mutated EV-DNA

**Figure 1:**
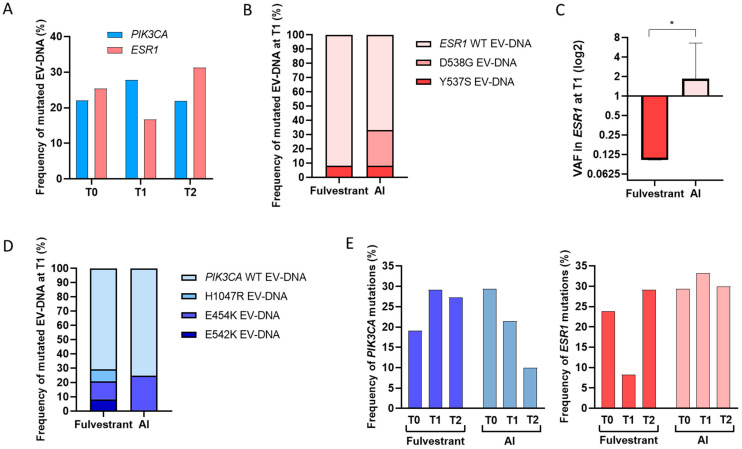
Distribution and dynamics of *ESR1* and *PIK3CA* mutations. Frequency of gene mutations at T0, T1, and T2 (**A**). Frequency of *ESR1* mutations for each codon according to study treatment (**B**). VAF mean and SD detected in *ESR1* at T1 according to study treatment (**C**). Frequency of specific *PIK3CA* mutations according to study treatment (**D**). Frequency of gene mutations at T0, T1, and T2 according to study treatment (**E**). * *p* < 0.05.

In addition to baseline samples from the 59 patients, 36 patients (61%) provided on-treatment samples 8 ± 2 weeks after starting ET ± additional targeted agents (T1), and 32 patients (54%) provided samples at the time of disease progression (T2). The evolution of mutational status in EV-DNA is depicted in Figure 1 and Supplementary Materials Figure S2.

*ESR1* mutation prevalence decreased from baseline to T1 (16.7%), while *PIK3CA* mutations increased to 27.8%, mainly due to a rise in the E545K mutation (Figure 1A). When patients were grouped by study treatment, a higher proportion of *ESR1* mutations was observed at T1 in those treated with AI compared to those treated with fulvestrant (*p* = 0.058; Figure 1B). Additionally, the mean VAF at T1 was 1.838% for those treated with AI and 0.103% for patients treated with fulvestrant (*p* = 0.029; Figure 1C).

In fulvestrant-treated patients, three cases (12.5%) positive for the D538G alteration at baseline became negative at T1, while the Y537S alteration showed variability: one case became negative at T1, one remained positive, and another was negative at T0 but became positive at T1. Therefore, Y537S was the only *ESR1* mutation in EV-DNA detected at T1 in patients treated with fulvestrant.

In AI-treated patients, D538G was detected in four (33.3%) cases at baseline, with two showing an increase in VAF and the other two becoming negative at T1. Specifically, the single Y537S-positive case at baseline treated with AI showed an increase in VAF from 5.3% at T0 to 16.6% at T1, with disease progression occurring one month after treatment initiation.

No significant associations were found between specific *PIK3CA* mutations at T1 and treatment type (Figure 1D). Notably, all *PIK3CA* mutations at T1 in the fulvestrant-treated group were detected when CDK4/6i were not included in the treatment regimen between T0 and T2.

The frequency of *ESR1* mutations in EV-DNA increased from 25.4% at baseline to 31.3% at progression, whereas the frequency of *PIK3CA* mutations remained stable at 22% from baseline through progression (Figure 1A). No significant associations were found between mutation status at T2 and shorter time to progression. Interestingly, when dynamic changes from T0 to T2 were evaluated by treatment type, the frequency of *PIK3CA* mutations decreased in the AI-treated group and increased in the fulvestrant-treated group, which showed the opposite trend to *ESR1* mutations (Figure 1E).

### 2.4. Comparative Analysis of EV-DNA and ctDNA

**Figure 2:**
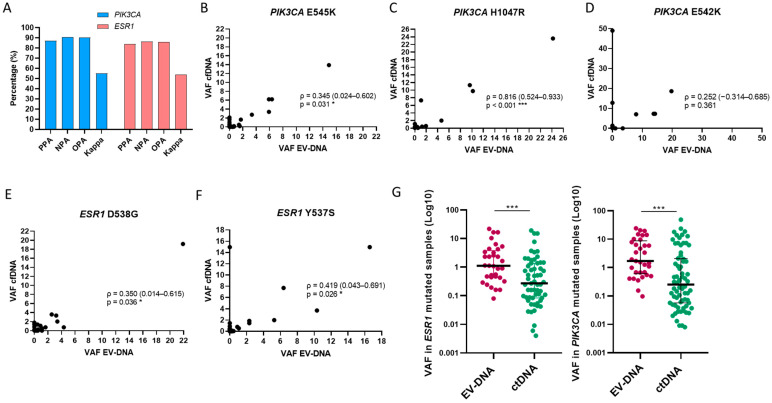
Concordance and correlation between EV-DNA and ctDNA. (**A**) Concordance of all digital PCR assessments for *PIK3CA* and *ESR1* genes. PPA, positive percentage agreement; NPA, negative percentage agreement; OPA, overall percentage agreement; Kappa, Cohen’s kappa coefficient. Correlation of VAFs between EV-DNA and ctDNA for each *PIK3CA* mutation (**B**–**D**). Correlation of VAFs between EV-DNA and ctDNA for each *ESR1* mutation (**E**,**F**). Comparison of VAFs between ctDNA and EV-DNA in mutated cases (**G**). *** *p* < 0.001.

Mutational data obtained from both EV-DNA and ctDNA were used to perform concordance analysis across all samples, time points, and alterations (Supplementary Materials Figure S3). The overall concordance rates for *PIK3CA* and *ESR1* mutations were 90% and 85%, respectively. Specifically, the positive percentage agreements (PPA) were 87% for *PIK3CA* and 83% for *ESR1*, while the negative percentage agreements (NPA) were 90% and 86%, respectively (Figure 2A). The correlation between the VAFs detected in EV-DNA and ctDNA for each mutation was also analyzed, revealing a moderate but statistically significant correlation for all alterations except E542K (Figure 2B–E). When only mutated cases were considered, a significant increase in VAF was observed in EV-DNA compared to ctDNA (*p* < 0.001 in both genes; Figure 2F).

**Figure 3:**
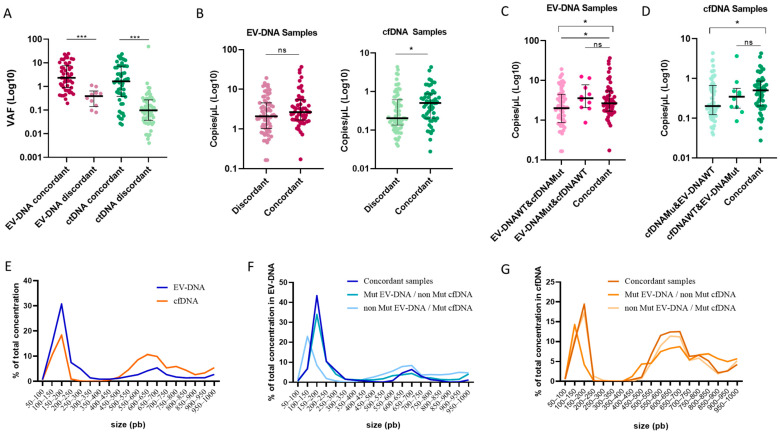
Comparison of concordant and discordant results between EV-DNA and ctDNA. VAFs and cop/µL distribution in concordant samples compared to discordant samples (**A**,**B**). Differences in cop/µL based on discordancy type and concordance (**C**,**D**). Percentage of total concentration for each size range in: EV-DNA and cfDNA (**E**), EV-DNA in concordant and discordant samples (**F**), cfDNA in concordant and discordant samples (**G**). * *p* < 0.05, *** *p* < 0.001, ns = not significant.

To explore potential reasons for discordance between EV-DNA and ctDNA results, we evaluated both VAF and total copies per microliter (cop/µL) detected in the dPCR analysis from each DNA source. In both EV-DNA and ctDNA analyses, mutated cases showed higher VAFs in concordant samples compared to discordant samples (*p* < 0.001 for both analyses; Figure 3A). Additionally, in discordant samples, a significant increase in VAF was observed in EV-DNA compared to ctDNA (*p* = 0.006). However, no significant differences in VAF were detected between EV-DNA and ctDNA in concordant samples. Our findings also indicated a trend towards higher cop/µL in EV-DNA samples with concordant mutations, with a significant increase observed in ctDNA samples (*p* = 0.037; Figure 3B).

When analyzing EV-DNA samples categorizing the types of discordance (Figure 3C), we observed a significant difference between discordant plasma samples with non-mutated EV-DNA and mutated ctDNA, and concordant plasma samples (*p* = 0.032), with the latter group exhibiting a higher concentration of cop/µL. The discordant samples with mutated EV-DNA and non-mutated ctDNA did not show a significant difference when compared to the concordant samples. In contrast, in the ctDNA analysis, discordant plasma samples where ctDNA was mutated and EV-DNA was wild-type showed a lower range of cop/µL and a significant difference when compared to the concordant samples (*p* = 0.04; Figure 3D).

We also analyzed the fragment sizes detected in both DNA sources in a subset of plasma samples (N = 9), and their distribution based on concordance results. In both EV-DNA and cfDNA, the most represented fragment size was 150–200 pb. However, approximately 60% of the DNA concentration was found between 100 and 250 pb in EV-DNA, whereas cfDNA displayed an additional large peak at 550–700 pb (Figure 3E, Supplementary Materials Figure S4). In EV-DNA, DNA was more fragmented in the subgroup with discordant results, where EV-DNA was wild-type and cfDNA was mutated (Figure 3F). Similarly, DNA was more fragmented in cfDNA samples with discordant results, where cfDNA was wild-type, and EV-DNA was mutated (Figure 3G). Thus, potential false-negative results in both DNA sources could be influenced by the small DNA fragment size. 

### 2.5. Mutations in EV-DNA and ctDN, and Their Use as Biomarkers

**Figure 4:**
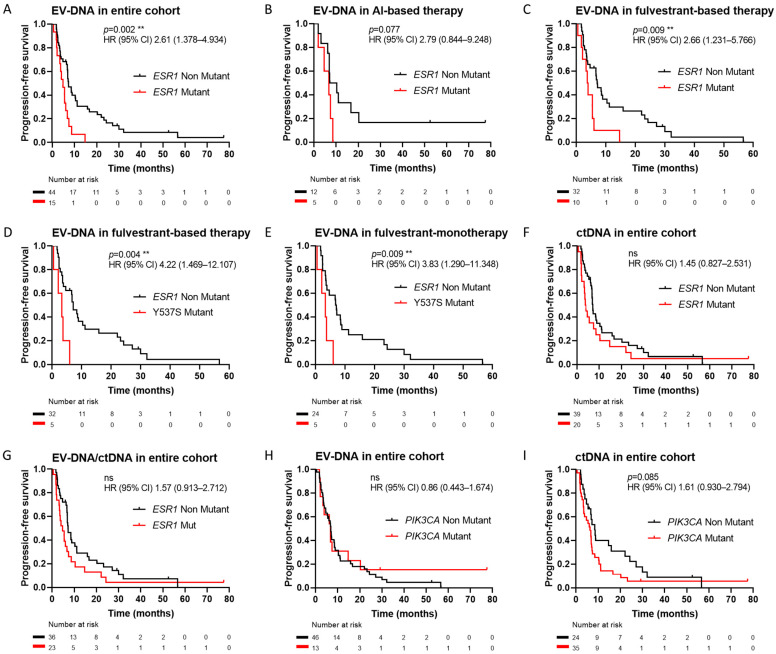
Prognostic value of *ESR1* and *PIK3CA* mutations at baseline. Prognostic value of *ESR1* mutations in EV-DNA in the entire cohort (**A**), in patients receiving AI-based therapy (**B**), and in patients receiving fulvestrant-based therapy (**C**). Prognostic value of Y537S mutation in EV-DNA in patients receiving fulvestrant-based therapy (**D**) and fulvestrant monotherapy (**E**). Prognostic value in the entire cohort of *ESR1* mutations in ctDNA (**F**) and combining EV-DNA and ctDNA (**G**). Prognostic value of *PIK3CA* mutations in EV-DNA (**H**) and ctDNA (**I**) in the entire cohort. ** *p* < 0.01, ns = not significant.

Baseline *ESR1* mutations in EV-DNA were significantly associated with a shorter PFS across the entire cohort (HR 2.61, 95% CI 1.378–4.934, *p* = 0.002; Figure 4A). This association was observed irrespective of the study treatment (AI-based therapy HR 2.79, 95% CI 0.844–9.248, *p* = 0.077, Figure 4B; fulvestrant-based therapy HR 2.66, 95% CI 1.231–5.766, *p* = 0.009, Figure 4C). In patients treated with fulvestrant, an analysis by alteration type showed a significant association only for the Y537S mutation (HR 4.22, 95% CI 1.469–12.107, *p* = 0.004; Figure 4D), with a median survival of 3.3 months compared to 5.4 months for D538G and 7.1 months for non-mutant cases. The prognostic significance of Y537S was also evident in patients not treated with CDK4/6i during the study (HR 3.83, 95% CI 1.290–11.348, *p* = 0.009; Figure 4E).

When the prognostic value of baseline *ESR1* mutations in ctDNA was assessed, no significant association with PFS was observed, either across the entire cohort (Figure 4F), by therapy type, or by alteration type (Supplementary Materials Figure S5A–C). Furthermore, *ESR1* mutations combining EV-DNA and ctDNA results were not significantly associated with shorter PFS (Figure 4G).

*PIK3CA* mutations in EV-DNA were not found to be associated with PFS in the overall cohort (*p* = 0.659; Figure 4H). It also showed no prognostic value in treatment-specific subgroups.

Conversely, a trend toward a significantly shorter PFS was found for patients with *PIK3CA* mutations in ctDNA in the entire cohort (HR 1.61, 95% CI 0.930–2.794, *p* = 0.085; Figure 4I). *PIK3CA* mutations were significantly prognostic in patients receiving fulvestrant monotherapy during the study (HR 2.35, 95% CI 0.820–6.735, *p* = 0.033; Supplementary Materials Figure S5D), with a median survival of 3.58 months for mutated and 7.10 months for non-mutant. However, this association was not maintained when CDK4/6i were added to fulvestrant (Supplementary Materials Figure S5E). In patients receiving AI-based therapy, no PFS differences were observed between the mutation status of *PIK3CA* in ctDNA (Supplementary Materials Figure S5F).

## 3. Discussion

This study followed a cohort of HR+/HER2-metastatic breast cancer patients who had previously been treated with AI and were subsequently given ET, with serial liquid biopsies collected throughout. This study provides valuable information on mutational profiling in two types of circulating tumor components, EV-DNA and ctDNA, that can support clinical decision-making.

Liquid biopsy is an emerging, minimally invasive technique in breast cancer management that has the potential to enhance personalized treatment strategies. Most studies to date have primarily focused on circulating tumor cells and ctDNA as the main tumor-derived materials analyzed through liquid biopsies [28,29]. In addition to these biomarkers, EVs isolated from breast cancer patients have been shown to contain distinct protein and RNA profiles with diagnostic, prognostic, and monitoring value [30,31,32]. Despite this, there is a lack of research exploring EV-derived DNA, highlighting a critical area for future investigation. In our study, *PIK3CA* and *ESR1* mutations were detected in EV-DNA in 22 and 25% of patients, respectively, with a distribution consistent with the patient population analyzed [33]. Our findings suggest that EV-DNA might represent a potential source of information regarding tumor mutational status. Although the literature on this subject is limited, tumor alterations, mainly in the *EGFR* and *KRAS* genes, have been detected in EV-derived DNA from biofluids such as plasma, pleural effusion, and cerebrospinal fluid [34]. To our knowledge, the study by Nakai M et al. is the only one analyzing EV-DNA in breast cancer, with a heterogeneous and limited number of samples across the three molecular subtypes (luminal, HER2+, and triple-negative breast cancer) [35]. Nakai et al. detected *PIK3CA* mutations in exosome-derived DNA in 8% of metastatic luminal A patients. This study demonstrated that the combination of EV-DNA and ctDNA yielded greater sensitivity in detecting mutations than either biomarker individually. In our study, the percentage of both mutated *PIK3CA* and *ESR1* samples was higher than in Nakai’s study, although the analyzed cohorts are not directly comparable. Our study focused exclusively on HR+/HER2-mBC patients who had progressed after prior AI treatment, suggesting an enrichment of *ESR1*-mutated clones in these tumors.

Interestingly, our analysis of EV-DNA revealed enrichment of *PIK3CA* mutations in patients with bone-only metastases, while *ESR1* mutations were enriched in those with visceral metastases. A higher frequency of *ESR1* mutations in solid tumors from patients with liver metastasis has been reported elsewhere [36]. Moreover, in a previous study analyzing ctDNA in this patient population [27], a trend toward a significant association was observed between mutated *PIK3CA* and non-visceral metastatic locations, but no associations were found for mutated *ESR1*. Therefore, EVs might provide distinct information regarding *ESR1* status compared to ctDNA, likely due to their differing origins. ctDNA is derived from all components detected in plasma, including non-vesicular extracellular particles and EVs, which are as different as nanovesicles and apoptotic bodies. Processes including necrosis, apoptosis, and active secretion are sources of ctDNA. However, variability was lower in our EV fraction, which was composed of small EVs described, at least in large part, as an active mechanism of intercellular communication [18]. Although there are few studies on EV-DNA, mRNA and non-coding RNA (ncRNA) loaded in EVs have been associated with poor prognosis and resistance to treatments such as tamoxifen [37,38,39,40]. Therefore, based on existing knowledge about EVs as mechanisms of resistance transfer and our current results, EV-DNA may serve as a valuable source of information for detecting biomarkers of resistance to ET.

Activating mutations in *ESR1* are acquired under selective pressure from AI and are rarely found in treatment-naive tumors (<5%); however, the detection percentage increases up to 40% in previously treated patients, particularly those treated with AI [41]. In our cohort, no significant association was found between the frequency of *ESR1* mutations in EV-DNA and greater previous exposure to ET. The homogeneity of our series, which consisted entirely of metastatic luminal patients previously treated with ET, might have contributed to the difficulty in identifying significant differences. Regarding dynamic evolution, we observed an increase in the number of cases with *ESR1* mutation in EV-DNA at progression. This result aligns with the literature, as these alterations are related to resistance to ET and may indicate enrichment due to clonal selection under treatment pressure. However, when evaluating T1, the frequency of *ESR1* mutations was lower than at baseline. Given that *ESR1* alterations confer resistance, this finding was unexpected as the mutated clones would typically be resistant. This decrease might be attributed to the varying impacts of the treatments administered. While *ESR1* mutations have been shown to predict resistance to AI clearly, the effects of fulvestrant on these mutations are more controversial [6]. In our cohort, when analyzing T1 samples based on treatment with fulvestrant or AI, we observed a higher proportion of *ESR1* mutations and higher VAFs in the AI-treated group compared to those treated with fulvestrant. However, the limited number of AI-treated patients in our series may have reduced statistical power in that subgroup, so worse prognostic was only weakly associated with mutations in EV-DNA and not in ctDNA, which has already been described [27]. Recently, it has been noted that the D538G and Y537S alterations in *ESR1* exhibit varying degrees of estrogen-independent ER activity [40]. Specifically, tumors with Y537S mutation have been described as being less sensitive to fulvestrant compared to other alterations in this gene [6,42]. Our findings are consistent with prior reports suggesting that tumors harboring D538G mutations are more sensitive to fulvestrant than those with Y537S. In our series, EV-DNA might provide a more efficient tumor source than ctDNA to evaluate the prognostic role of *ESR1* mutations. In clinical practice, it is common to switch AI-refractory patients to fulvestrant. In these cases, monitoring Y537S in EV-DNA could offer valuable insights for improved disease management.

Activating mutations in *PIK3CA* are a common truncal genomic alteration in HR+/HER2-metastatic breast cancer. The development of sub clonal mutations under antiestrogen therapies is relatively rare [33]. Poor outcomes in patients with mutant PIK3CA treated with fulvestrant compared to those with wild-type *PIK3CA* have been reported, but in the context of AI, mutated *PIK3CA* has been linked to a longer PFS [43,44]. Our findings align with previous studies, where the dynamic evolution of *PIK3CA* mutation frequencies varied with the treatment received, showing an increase in the cohort treated with fulvestrant and a decrease in those treated with AI. Recent studies in *PIK3CA*-mutant populations have shown that the addition of alpelisib, a selective PI3K inhibitor, or CDK4/6 inhibitors to fulvestrant significantly improves PFS [45]. In our study, *PIK3CA* mutations at baseline were found prognostic in ctDNA but not in EV-DNA, both across the entire cohort and specifically in patients treated with fulvestrant monotherapy.

We also compared the mutational status in EV-DNA and ctDNA, finding overall high concordance rates for *PIK3CA* and *ESR1*. Higher VAFs were found in EV-DNA compared to ctDNA, which may be attributed to a lower amount of wild-type DNA released by normal cells in the case of EVs. In contrast, a proportion of cfDNA originates from non-tumor cells, such as large ectosomes and part of apoptotic bodies [18]. A more detailed analysis identified factors that may influence the discordant cases detected between EV-DNA and ctDNA. Lower VAFs were observed in discordant samples for both analyses, which complicates detection as these samples may be close to the technique’s detection limit. Interestingly, potential false-negative results in EV-DNA (wild-type EV-DNA and mutated ctDNA) showed lower cop/µL levels in EV-DNA analysis, suggesting that the quantity of cop/µL may be insufficient to detect mutations with low VAF, possibly due to limited tumor material in EV-DNA or reduced vesicle release. However, potential false-negative results in ctDNA (mutated EV-DNA and wild-type ctDNA) did not show lower cop/µL levels compared to concordant samples. This lack of difference may indicate a higher wild-type background derived from non-tumor cells, decreasing the VAF of mutations and making it difficult to detect. Finally, a different size distribution was observed between EV-DNA and ctDNA, with more fragmented DNA detected in cases with potential false-negative results in both sources. These smaller fragments may hinder amplification during dPCR. To facilitate proper amplification, primers for smaller amplicons should be designed.

In summary, our results suggest that EV-DNA might be a distinct source of tumor DNA that may complement conventional liquid biopsy ctDNA. Specifically, *ESR1* mutations in EV-DNA may serve as better prognostic biomarkers compared to those detected in ctDNA, whereas *PIK3CA* alterations only showed an association with poor outcomes in ctDNA. This difference may be explained by the distinct origins of EV-DNA and ctDNA, as discussed above. Alterations occurring early in tumorigenesis, which are present across many tumor clones, may be better captured in ctDNA, while resistance-associated alterations may be more effectively detected in EV-DNA. While the mutation frequencies for *PIK3CA* and *ESR1* in EV-DNA observed in this study are consistent with the prevalence reported in the literature, the limited sample size may affect the statistical power to draw definitive conclusions. Our study focused on the most common *ESR1* point mutations. However, missense mutations have been identified at more than 51 additional residues, predominantly within the ligand-binding domain (LBD). Among these, the most frequently observed mutations, such as Y537 and D538, are characterized as hormone-independent activating mutations, whereas others, such as K303R and E380Q, enhance estrogen sensitivity [46]. In addition, other less common mechanisms that confer secondary endocrine resistance should also be evaluated, including *ESR1* fusions and ER expression loss due to epigenetic changes [41]. Future studies with larger sample sizes and employing broader genomic approaches are needed to confirm these findings and identify additional alterations and resistance mechanisms, allowing for a deeper characterization of the role of EV-DNA in assessing specific *ESR1* alterations based on treatment type. Another key factor affecting future results on biomarkers is the continuous evolution of treatment strategies, which now often include ET combined with targeted therapies like CDK4/6 and PI3K inhibitors. Additionally, the novel SERD elacestrant in patients with detectable *ESR1* mutations in ctDNA, and a few others are currently under development, such as camizestrant [47]. Investigating *ESR1* mutations in EV-DNA as a prognostic and predictive biomarker could help optimize treatment selection and monitoring with novel SERDs in the upcoming treatment landscape of HR+/HER2-mBC.

## 4. Materials and Methods

### 4.1. Study Design

A prospective observational study was conducted involving a cohort of 59 HR+/HER2-mBC patients who had previously received treatment with AI in either the adjuvant or metastatic setting with a subsequent ET with either a SERD or an AI, either alone or in combination with additional targeted agents. Participants were enrolled in the study at the Breast Cancer Unit of the Hospital Clínico San Carlos from March 2016 to April 2021. Patients with symptomatic visceral involvement, life expectancy of less than six months, or significant comorbidities were excluded from the study. Two patients were also excluded because digital PCR amplification of their EV-DNA samples could not be obtained for any of the interrogated mutations. Plasma analysis did not influence patient management during the study period. The Institutional Ethics Committee of Hospital Clínico San Carlos approved this study (16/087-E_BD), which was conducted in accordance with the Good Clinical Practice Guidelines and the Declaration of Helsinki. All participants provided written informed consent prior to their inclusion in the study. Patients were followed up until September 2022. Primary resistance was defined as relapse within the initial 2-year period of adjuvant ET or the occurrence of progressive disease within the first 6 months of first-line ET in the metastatic context. Secondary resistance, on the other hand, was characterized by relapse while receiving adjuvant ET beyond 2 years of treatment, relapse within 1 year of completing adjuvant ET, or the manifestation of progressive disease after 6 months of ET in the metastatic setting [48]. The median follow-up period was determined from the date of T0 extraction to exit, loss to follow-up, or the end of the study, whichever occurred first. Progression-free survival (PFS) was defined as the time from treatment initiation to the first occurrence of disease progression or death from any cause (whichever occurs first).

### 4.2. Biological Samples Obtaining

Blood samples were collected at various time points during the study: prior to the initiation of ET with or without additional targeted agents (T0, baseline), 8 ± 2 weeks after the treatment began (T1), and at the time of disease progression (T2) for patients experiencing rapid disease progression. For sample collection, approximately 20 mL of blood was collected in EDTA tubes and centrifuged at 1900× *g* for 10 min at 4 °C within two hours of collection. The resulting plasma was then centrifuged at 16,000× *g* for 10 min at 4 °C. The plasma samples were aliquoted and stored at −80 °C until further processing for EVs isolation or cfDNA extraction. Four milliliters of plasma was used for ctDNA isolation and five hundred microliters for EVs isolation. Sufficient plasma was available to conduct the study on 59 T0 samples (baseline), 36 T1 samples (treatment), and 32 T2 samples (progression). 

### 4.3. Extracellular Vesicles Isolation 

EVs were isolated following the Thrombin Plasma Prep for Exosome Precipitation protocol (System Biosciences, Mountain View, CA, USA). According to it, 5 µL of thrombin was added to 500 µL of plasma. The plasma was then treated with 113.4 μL ExoQuick™ for 30 min at 4 °C. The samples were then centrifuged at 1500× *g* for 30 min. The supernatant was discarded, and the EVs-enriched pellet was resuspended in 1 mL of PBS. 

### 4.4. DNA Extraction 

To isolate EV-DNA, the QIAamp Circulating Nucleic Acid Kit (QIAGEN GmbH, Hilden, Germany) was used starting from 900 μL of PBS-resuspended EVs sample. The protocol was revised to increase the incubation time at 60 °C by up to 1 h. An eluate of 140 μL was obtained for each sample, which was concentrated utilizing SpeedVac™ vacuum concentrator (ThermoFisher, Carlsbad, CA, USA) to a final volume of 40 μL. The same protocol and kit were utilized to obtain the plasma ctDNA starting from 4 mL of plasma. The size of DNA fragments from EV-DNA and cfDNA was analyzed in 9 plasma samples (3 concordant samples, 3 discordant samples with mutated EV-DNA and wild-type cfDNA, and 3 discordant samples with wild-type EV-DNA and mutated ctDNA) by TapeStation (Agilent, Santa Clara, CA, USA).

### 4.5. Analysis of ESR1 and PIK3CA Mutations by dPCR

Isolated EV-DNA and ctDNA samples were analyzed using the QuantStudioTM 3D Digital PCR System (Thermo Fisher Scientific, Carlsbad, CA, USA) to detect alterations in *ESR1* (Y537S and D538G) and *PIK3CA* (E545K, E542K, and H1047R). Wet lab-validated TaqMan dPCR Liquid Biopsy assays were used for *PIK3CA* analysis, and TaqMan probes were designed for *ESR1* analysis using the Custom TaqMan Assay Design Tool (Thermo Fisher Scientific, Carlsbad, CA, USA). TaqMan probe information and dPCR conditions are shown in Supplementary Materials Table S1. When analyzing the chips, for a mutation to be considered present, two measurements yielding concordant results were required for each experiment. A quality threshold of 0.6 was applied. Variant allele frequency (VAF; percentage of mutant copies in relation to the total copies) and copies/µL (mutated cop/µL + nonmutated cop/µL) for each codon were analyzed. For each alteration, cop/µL were normalized with the median of series for this alteration and starting of plasma volume.

### 4.6. Statiscal Analysis

Continuous variables were summarized using mean, standard deviation, median, minimum, and maximum values. Categorical variables were described by their absolute and relative frequencies. For variable comparison, the χ^2^ test, Mann–Whitney test, and Kruskal–Wallis test were employed, depending on the variable type and distribution. Survival outcomes were estimated using the Kaplan–Meier method, and the Mantel–Cox log–rank test was applied to assess significant differences between survival curves. Statistical analyses and graph design were conducted using R software version 3.9 (https://cran.r-project.org/; accessed on 10 June 2024) and GraphPad Prism software version 10 (San Diego, CA, USA). Oncoprint visualizations were generated using OncoPrinter and MutationMapper tools from cBioportal (https://www.cbioportal.org/oncoprinter; accessed on 26 November 2024).

## Figures and Tables

**Table 1:** Clinicopathological characteristics of the patients according to mutational status at baseline.

Characteristics	All Patients	*PIK3CA* Mutant	*PIK3CA* Wild-Type	*p*-Value	*ESR1* Mutant	*ESR1* Wild-Type	*p*-Value
Age (median ± SD)	65.5 (39.1–87.7)	71.2 (47–86.55)	65.1 (39.1–87.7)	NA	68.3 (43.9–87.7)	62.7 (39.1–86.55)	NA
**Age Range (%)**							
≤65 yr >65 yr	26 (44) 33 (56)	4 (6.8) 9 (15.3)	22 (37.3) 24 (40.7)	0.274	3 (5.1) 1 (1.7)	23 (39.0) 21 (35.6)	0.029
**ECOG PS Scale (%)**							
0 1	56 (95) 3 (5)	12 (20.3) 1 (1.7)	44 (74.6) 2 (3.4)	0.627	13 (22.0) 2 (3.4)	43 (72.9) 1 (1.7)	0.092
**Line of endocrine therapy (%)**							
First Second Third or Higher	7 (12) 21 (36) 31 (52)	1 (1.7) 6 (10.2) 6 (10.2)	6 (10.2) 15 (25.4) 25 (42.4)	0.638	2 (3.4) 6 (10.2) 7 (11.9)	5 (8.5) 15 (25.4) 24 (40.7)	0.870
**Previous line of chemotherapy (%)**							
Yes No	40 (68) 19 (32)	8 (13.6) 5 (8.5)	32 (54.2) 14 (23.7)	0.584	8 (13.6) 7 (11.9)	32 (54.2) 12 (20.3)	0.165
**Previous CDK4/6 inhibitor (%)**							
Yes No	23 (39) 36 (61)	8 (13.6) 5 (8.5)	15 (25.4) 31 (52.5)	0.059	6 (10.2) 9 (15.3)	17 (28.8) 27 (45.8)	0.925
**Endocrine therapy (T0-T2) (%)**							
Aromatase Inhibitors Fulvestrant	17 (29) 42 (71)	5 (8.5) 8 (13.6)	12 (20.3) 34 (57.6)	NA	6 (10.2) 9 (15.3)	11 (18.6) 33 (55.9)	NA
**CDK4/6 inhibitor (T0-T2) (%)**							
Palbociclib Ribociclib None	8 (14) 6 (10) 45 (76)	3 (5.1) 0 (0) 10 (16.9)	5 (8.5) 6 (10.2) 35 (59.3)	NA	1 (1.7) 0 (0) 14 (23.7)	7 (11.9) 6 (10.2) 31 (52.5)	NA
**Metastatic sites (%)**							
Visceral Non-visceral Bone-only Non-bone-only	33 (56) 26 (44) 13 (22) 26 (44)	5 (8.5) 8 (13.6) 6 (10.2) 4 (6.8)	28 (47.5) 18 (30.5) 7 (11.9) 22 (37.3)	0.150 0.038	14 (23.7) 1 (1.7) 1 (1.7) 8 (13.6)	19 (32.2) 25 (42.4) 12 (20.3) 18 (30.5)	<0.001 0.106
***De novo*** **Metastases (%)**							
Yes No	13 (22) 46 (78)	4 (6.8) 9 (15.3)	9 (15.3) 37 (62.7)	0.389	5 (8.5) 10 (16.9)	8 (13.6) 36 (61.0)	0.221
**Endocrine resistance (%) ^1^**							
Sensitive Primary Secondary	19 (32) 17 (29) 15 (25)	4 (6.8) 4 (6.8) 2 (3.4)	15 (25.4) 13 (22.0) 13 (22.0)	0.753	5 (8.5) 4 (6.8) 3 (5.1)	14 (23.7) 13 (22.0) 12 (20.3)	0.911
**Primary tumor histology (%)**							
Ductal Lobular Other (NOS)	38 (65) 5 (8) 10 (17)	6 (10.2) 2 (3.4) 3 (5.1)	32 (54.2) 3 (5.1) 7 (11.9)	0.330	9 (15.3) 2 (3.4) 3 (5.1)	29 (49.2) 3 (5.1) 7 (11.9)	0.709
**Primary tumor grade (%)**							
Low Intermediate High	3 (5) 35 (59) 10 (17)	1 (1.7) 10 (16.9) 1 (1.7)	2 (3.4) 25 (42.4) 9 (15.3)	0.460	0 (0) 9 (15.3) 4 (6.8)	3 (5.1) 26 (44.1) 6 (10.2)	0.369
**Primary tumor Ki67 (%)**							
1–14 15–20 >20	23 (39) 9 (15) 13 (22)	8 (13.6) 1 (1.7) 2 (3.4)	15 (25.4) 8 (13.6) 11 (18.6)	0.249	7 (11.9) 2 (3.4) 4 (6.8)	16 (27.1) 7 (11.9) 9 (15.3)	0.885
**HER2 status (%)**							
IHC (+2), FISH (−) IHC (+1), FISH (−) IHC (0)	18 (31) 17 (29) 16 (27)	4 (6.8) 5 (8.5) 3 (5.1)	14 (23.7) 12 (20.3) 13 (22.0)	0.760	1 (1.7) 7 (11.9) 5 (8.5)	17 (23.7) 10 (16.9) 11 (18.6)	0.044

^1^ As per ESMO criteria. Abbreviations: ECOG PS: Eastern Cooperative Oncology Group Performance Status. IHC: immunohistochemistry. Unknown values were not computed in statistical analyses.

## Data Availability

The data generated and analyzed in this study are included in the article and the Supplementary Materials. Anonymized individual genomic and clinical data can be made available for academic purposes only upon reasonable request to the corresponding author, subject to a data transfer agreement and approval from the Ethics Committee.

## Supplementary Materials

The following supporting information can be downloaded at https://www.mdpi.com/article/10.3390/ijms252313045/s1.
