# Supplementary material for: Molecular Profiling of Endocrine Resistance in HR+/HER2-Metastatic Breast Cancer: Insights from Extracellular Vesicles-Derived DNA and ctDNA in Liquid Biopsies
Source: Int J Mol Sci. 2024 Dec 4;25(23):13045. doi: 10.3390/ijms252313045 (PMC11641624; doi:10.3390/ijms252313045)
Supplement: Supplementary file 1 [file ijms-25-13045-s001.zip › ijms-3318849-supplementary/Supplementary Figures.pdf]

Supplementary Figures

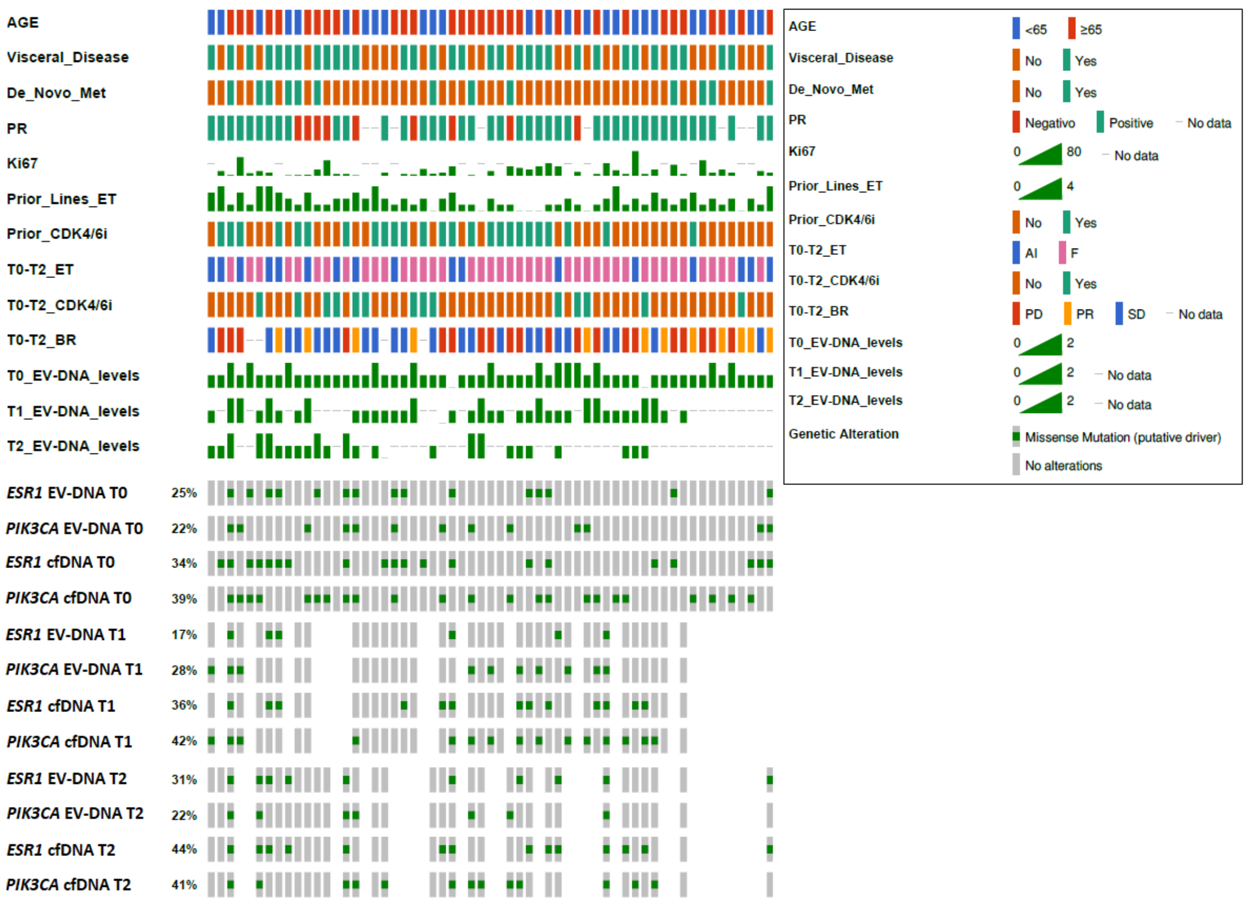

Supplementary Figure S1. Oncoprint plot. Somatic alterations and clinical–pathological characteristics per patient. De_Novo_Met: Patients that presented *de novo* mBC at diagnosis. PR: Progesterone Receptor at diagnosis. Ki67: percentage of Ki67expression at diagnosis. Prior_ET_Lines: number of lines of endocrine therapy received before T0. T0-T2_BR: Best response achieved between T0 and T2.

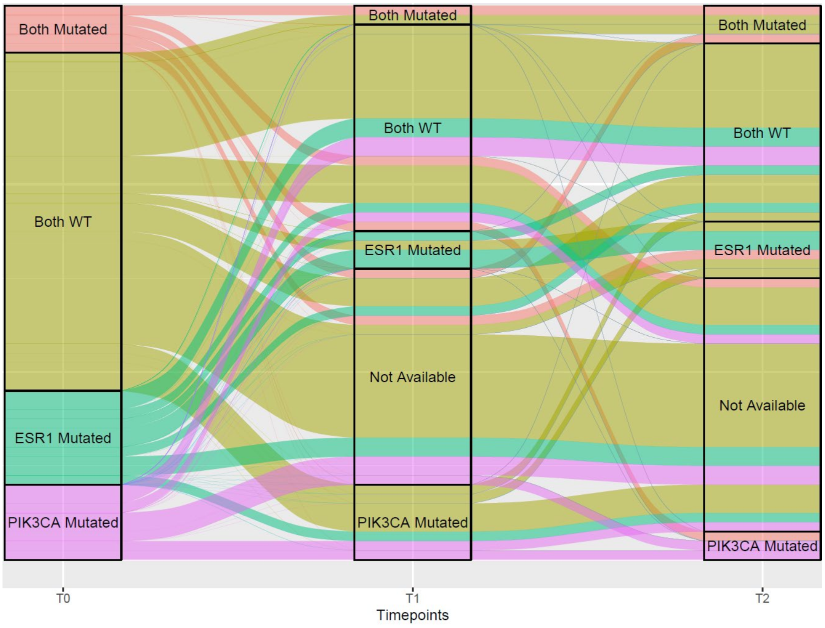

Supplementary Figure S2. Alluvial diagram. Mutational dynamics between T0 and T2 time points. Samples that were either not collected during the study or depleted during the prior analysis focused on cfDNA assessment are classified as “Not Available”.

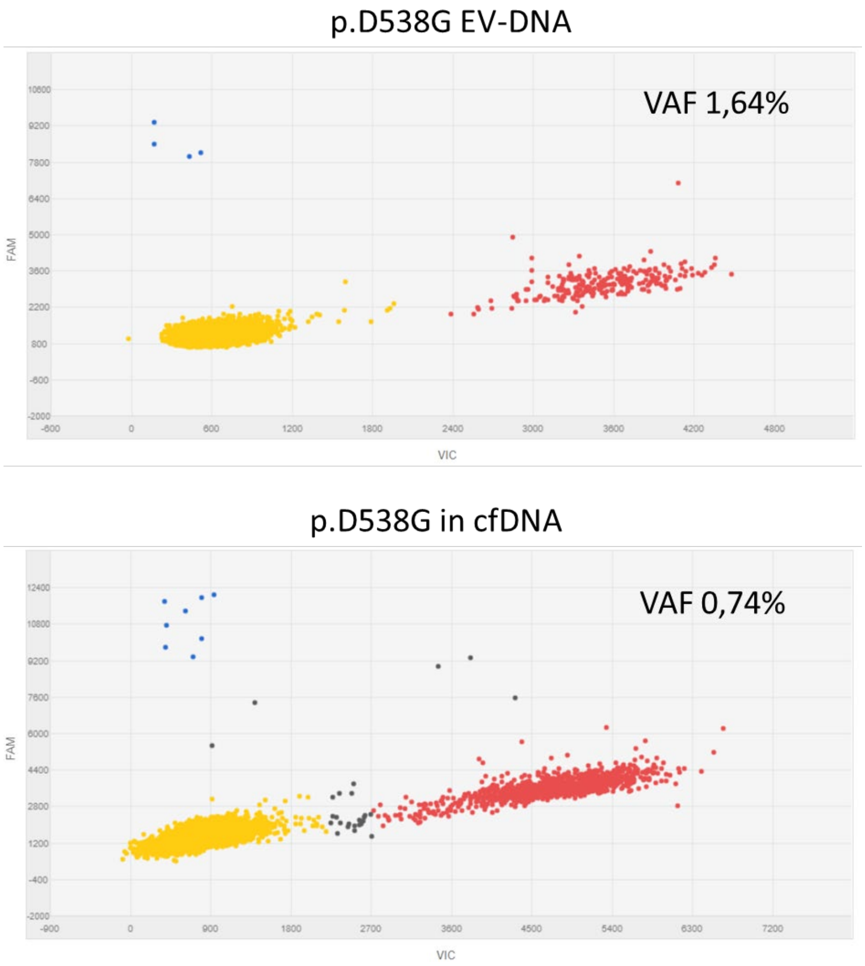

Supplementary Figure S3. Digital PCR results. Examples of detection of the ESR1 mutations in EV-DNA (A) and cfDNA (B) using digital PCR

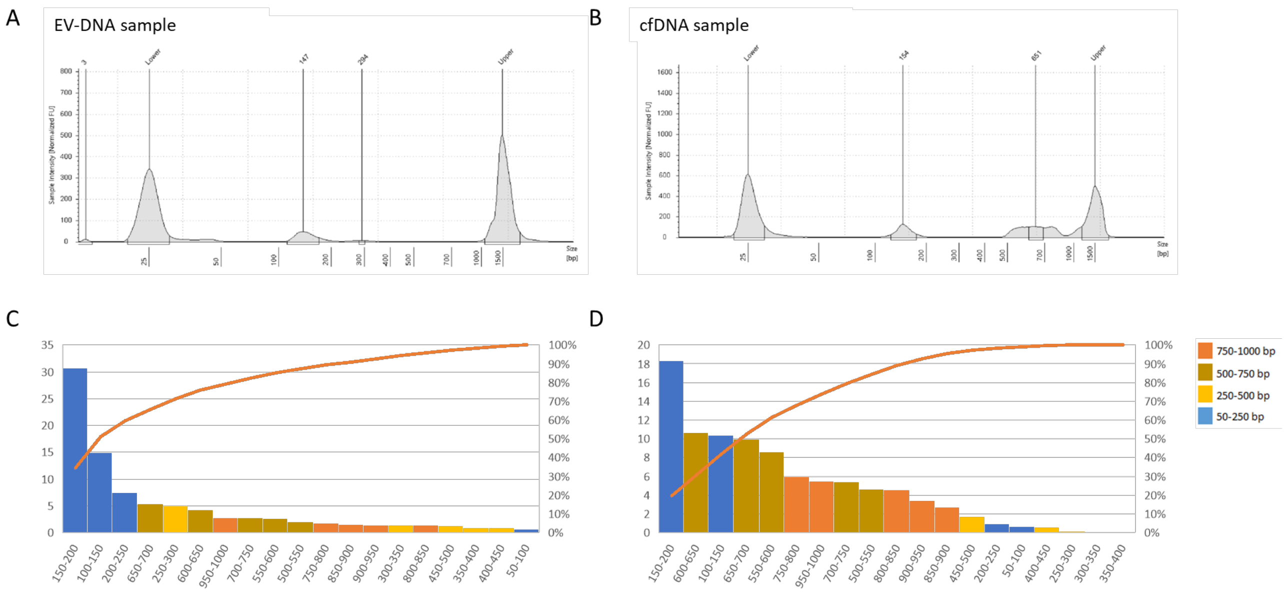

Supplementary Figure S4. Sizes of fragments detected in EV-DNA and cfDNA. Examples of size distribution of EV-DNA (A) and cfDNA (B) using TapeStation system. Frequency and cumulative percentage of DNA concentration for each size range in EV-DNA (A) and cfDNA (B).

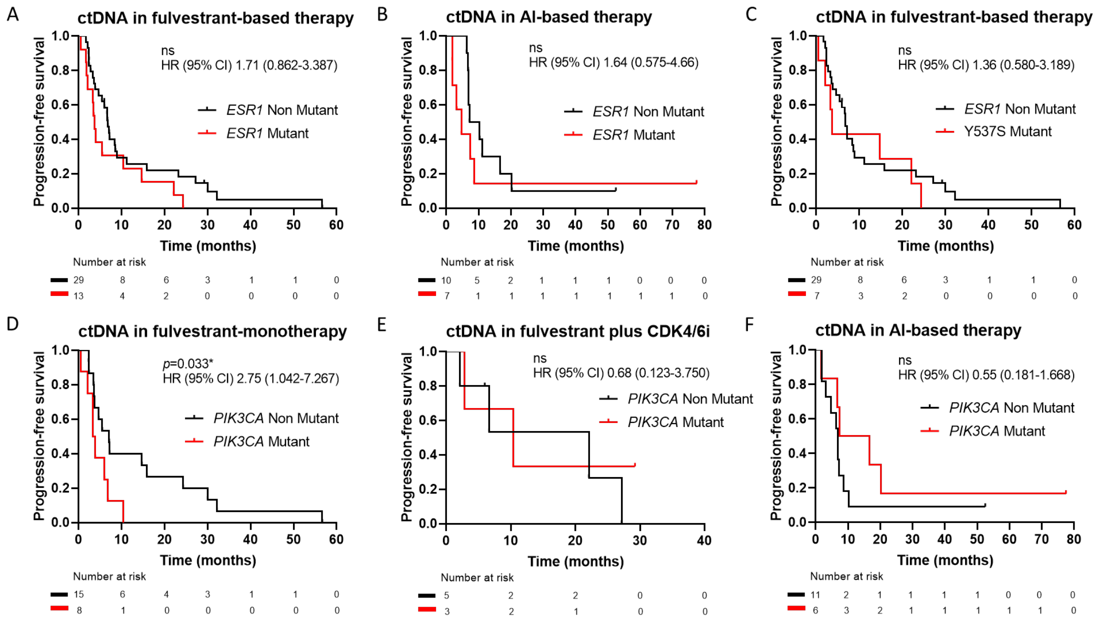

Supplementary Figure S5. Prognostic value of *ESR1* mutations in ctDNA in patients receiving fulvestrant- and AI-based therapy (A and B, respectively). Prognostic value of Y537S mutation in ctDNA in patients receiving fulvestrant-based therapy (C). Prognostic value of *PIK3CA* mutations in ctDNA in patients receiving fulvestrant monotherapy (D), fulvestrant plus CDK4/6 inhibitors (E) and AI-based therapy (F).
